# A neural network model for development of reaching and pointing based on the interaction of forward and inverse transformations

**DOI:** 10.1111/desc.12565

**Published:** 2017-06-20

**Authors:** Naohiro Takemura, Toshio Inui, Takao Fukui

**Affiliations:** ^1^ Department of Intelligence Science and Technology Graduate School of Informatics Kyoto University Japan; ^2^ Present address: Department of Psychology Otemon Gakuin University Ibaraki Osaka Japan; ^3^ Present address: Faculty of System Design Tokyo Metropolitan University Hino Tokyo Japan

## Abstract

Pointing is one of the communicative actions that infants acquire during their first year of life. Based on a hypothesis that early pointing is triggered by emergent reaching behavior toward objects placed at out‐of‐reach distances, we proposed a neural network model that acquires reaching without explicit representation of ‘targets’. The proposed model controls a two‐joint arm in a horizontal plane, and it learns a loop of internal forward and inverse transformations; the former predicts the visual feedback of hand position and the latter generates motor commands from the visual input through random generation of the motor commands. In the proposed model, the motor output and visual input were represented by broadly tuned neural units. Even though explicit ‘targets’ were not presented during learning, the simulation successfully generated reaching toward visually presented objects at within‐reach and out‐of‐reach distances.

## RESEARCH HIGHLIGHTS

- We hypothesized that infants’ primitive pointing behavior emerges as a nonsocial‐orienting action based on the acquisition of visuo‐motor transformations through motor babbling.
- We proposed a neural network model for the development of reaching and pointing based on visuo‐motor transformations.
- Through random arm movements (motor babbling), the network learned both forward (motor‐to‐vision) and inverse (vision‐to‐motor) transformations without the presentation of an explicit target for reaching.
- Although the network only learned these transformations for random movements, the loop of the forward and inverse transformations can generate successive motor commands to reach toward visually presented objects, thereby implementing a feedback control with an internal estimation.
- The model can also simulate the infants’ primitive pointing behavior toward objects that are placed out of reach.

## 1 INTRODUCTION

### 1.1 Reaching and pointing development in infants

Human beings have the ability to communicate with each other using both language and symbolic gestures. As for symbolic gestures, even 1‐year‐old infants use reaching and pointing in different social contexts. Reaching is used in the imperative‐instrumental context, and pointing is used in the declarative‐referential context (Franco & Butterworth, 1996). Tomasello, Carpenter, and Liszkowski (2007) have argued that pointing is an action that already exhibits a variety of communicative intentions even when performed by 1‐year‐old infants.

However, it is still unclear what developmental route mediates the acquisition of pointing that has a social function. One possibility is that infants first perform pointing as an individual action that has no communicative intention, but, subsequently, they acquire the social context of their action from the responses of adults (Carpendale & Carpendale, 2010). Specifically, the adults’ interpretations of the infants’ actions help infants construct the association between their orienting behaviors, where they extend their arm toward a target object and out‐of‐reach distances, and their social contexts.

Before infants begin to engage in orienting behavior at around 9 months (Carpendale & Carpendale, 2010), they begin to reach toward visually presented targets at 3–5 months of age (von Hofsten, 1984). As a midpoint between reaching toward visual targets and communicative pointing, ‘pointing‐like’ movement, as a nonsocial orienting action (Carpendale & Carpendale, 2010), is indispensable for an infant's developmental trajectory. The present study focuses on how this nonsocial orienting action emerges as a form of reaching toward a visual stimulus. Specifically, this study aims to elucidate the computational mechanisms that inform the emergence of this nonsocial action (i.e., extending one's arm toward a target object located at an out‐of‐reach distance without social intention); it also seeks to identify how those mechanisms work.

### 1.2 Feedback control of reaching

Infants monitor their hands during movement and they correct their hand trajectory against motor deviation or the target movement when performing visually guided reaching. Visually guided reaching is controlled by motor commands that are generated online from the current hand position and the target position. This feedback control using sensory‐to‐motor transformation is the basis for visually guided reaching. However, Bushnell (1985) showed that infants perform successful reaching without online visual feedback of their hand after 7 months of age. The importance of visual feedback of the hand decreases, and the movement becomes smoother and more predictive. Reaching in the dark also develops at the same time as reaching in the light, at around the age of 4 months (Clifton, Muir, Ashmead, & Clarkson, 1993).

Feedback motor control in the human body is innately capable of incorporating with uncertainty in order to compensate for the neural noise and delay that interfere with the sensory and motor paths (van Beers, Baraduc, & Wolpert, 2002; Wolpert, Ghahramani, & Jordan, 1995). The idea of an internal forward model has been developed to compensate for this noise and delay. The forward model predicts sensory feedback of the hand from the motor commands that have been generated, and then this prediction is used as an alternative feedback signal to generate the next motor command (e.g., Miall, Weir, Wolpert, & Stein, 1993; Todorov & Jordan, 2002). This type of control mechanism is called *model predictive control* (Maciejowski, 2000).

**Figure 1:**
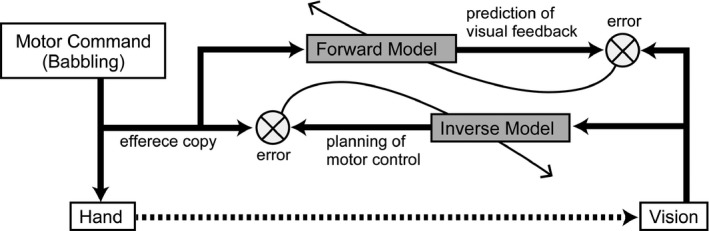
Schematic view of model. The forward model predicts visual feedback from an efference copy of the motor command, and learns their relationship through the error between predicted and actual vision. The inverse model generates the motor plan from visual input, and learns their relationship through the error between planned and actual motor output

*Model predictive control* requires two types of sensory‐to‐motor transformations (internal models): forward and inverse transformations (see Figure 1). A forward transformation (forward model) predicts the resultant sensory feedback of a motor command while an inverse transformation (inverse model) generates a motor command necessary for the resultant (required) sensory signal. A forward transformation is important for solving problems in the feedback motor control. In order to generate motor commands to achieve a desired state of the body and environment, the information about the ‘current’ state is necessary. However, the sensory signal arrives at the central nervous system (CNS) with delay and noise. The CNS uses a forward transformation to estimate the current state before the sensory feedback arrives (Ogawa, Inui, & Sugio, 2007) with an efference copy (or reafference: von Holst & Mittelstaedt, 1971) of the motor command. Then, the difference between the estimated and actual sensory feedback (prediction error) is used to update the current estimation (Wolpert et al., 1995). The loop of two sensorimotor transformations allows the internal feedback control to achieve a reaching movement (Desmurget & Grafton, 2000). Less dependence on vision during reaching (Bushnell, 1985; Clifton et al., 1993) suggests that infants are able to use forward prediction at the age of 7 months. During the period of 4–7 months of age, it seems that infants acquire reaching control using forward and inverse transformations. We assume that an infant's development of reaching is achieved by learning these two sensorimotor transformations.

### 1.3 Neural network model for reaching development

Bullock, Grossberg, and Guenther (1993) proposed the DIRECT (DIrection‐to‐Rotation Effector Control Transform) model, a self‐organizing neural model for eye–hand coordination in which the learning of spatial‐to‐motor and motor‐to‐spatial transformations is based on *motor babbling* (spontaneous random hand movement). Infants at 2–3 months of age persistently look at their hands (hand regard) (White, Castle, & Held, 1964). From this continuous observation of hand movement, the neural network simultaneously learns both the forward (motor‐to‐spatial) and the inverse (spatial‐to‐motor) mapping of the movement. However, the DIRECT model explicitly requires a calculation of the difference between the current hand position and the target position, implying explicit target representation during learning, and it does not consider how this calculation is acquired. The DIRECT model cannot simulate the reaching action towards a target at an out‐of‐reach distance because it has to learn a non‐existent reaching path.

Instead, we assumed that infants do not learn the entire movement path to achieve reaching; rather, they learn forward and inverse transformations for small components of the movement through motor babbling. In order to achieve reaching, they need to ‘connect’ these small movements, but they do not learn how to connect them. Here, we propose a mechanism to generate a succession of small movements by assuming that the representations of the visual position and the motor command are ambiguous so that the broad relationship between these representations can be acquired. The model only generates a motor command that does not accomplish bringing the hand to the required visual position in one shot, but the loop of the forward and inverse transformations continuously generates the motor commands of emergent reaching toward the position. By this mechanism, infants can achieve an unexperienced reaching movement even if they reach toward an out‐of‐reach position.

**Figure 2:**
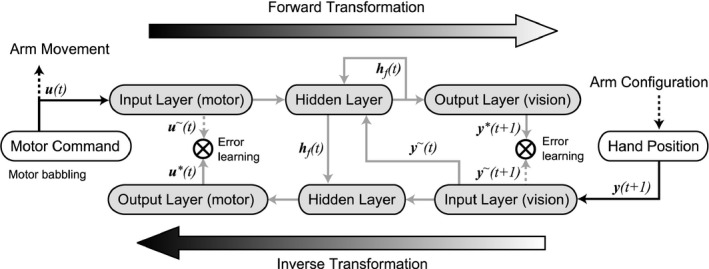
Neural network structure. The network consists of forward transformation and inverse transformation. Black solid arrows and gray solid arrows denote neural encoding and neural connections, respectively. Gray dashed lines indicate teacher signals for learning. Black dashed lines signify interaction with the physical arm. In the forward transformation, the neural representation of motor command *u*(*t*) and visual feedback of hand position ***y***(*t*), i.e., ***u*** ^*~*^(*t*) and ***y*** ^*~*^(*t*), respectively, are input, and the prediction of the next visual feedback ***y*** *(t + 1) is output. The hidden layer has a recurrent connection. In the inverse transformation, the neural representation of visual feedback ***y*** ^*~*^ *(t + 1)* and the activation of the hidden layer in the forward transformation ***h***_*f*_(*t*) are input, and the ‘postdiction’ of motor command ***u*** *(*t*) is output

In the present study, we propose a neural network model (Figure 2) to explain the emergence of reaching and primitive pointing actions. The model has the following features:

1. Infants first acquire forward and inverse visuo‐motor transformations of hand movements through their observation of random movement (motor babbling). Specifically, the forward transformation learns the resultant visual hand position of a random motor command with a simple recurrent network where an internal representation of the visual hand position before the movement is held in the hidden layer, and the inverse transformation learns the required motor command that brings the hand from the visual position before the movement to the position after the movement.
2. The visual hand position before the movement is internally represented in the hidden layer of the forward network, and the visual hand position after the movement is visually input. A feedback control using an internal estimation autonomously develops by connecting the hidden layer of the forward transformation to the hidden layer of the inverse transformation.
3. The motor command and the visual feedback are ambiguous. Specifically, these signals are represented in a ‘place code’ (for review, see Sanger, 2003) by populations of broadly tuned neural units. This enables the neural units to react to the signals that are not preferred, making it possible to reach toward the out‐of‐reach distance even though motor babbling is a movement that occurs in the within‐reach distance, and the movement toward an out‐of‐reach position is not experienced during learning.

### 1.4 Simulating reaching and pointing

After learning, we tested the capacity of the network to perform the forward and inverse transformations within one step of a motor command. Subsequently, the simulations of reaching toward targets presented in the within‐reach distance and the out‐of‐reach distance were performed. The proposed mechanism explains the emergence of infant primitive pointing (or reaching) without communicative intentions. The behavior is a mere ‘reaction’ that is not driven by internal intention. The network does not learn how to reach toward a ‘target’; it only acquires the forward and inverse transformations through motor babbling. In the simulation of reaching and pointing, the input of the representation of a visual position instead of a hand position drives the hand toward that location, without the need for any mechanism of intention. To avoid the model's confusion by simultaneous visual representation of the hand and the target, the hand vision was removed during reaching and pointing.

## 2 MODEL

A neural network model is proposed that learns the forward and inverse transformations for two‐joint arm movements. The network learns the relationship of the motor command and the hand position through the visual observation of the hand moving randomly (Figure 1). Both the motor command and the visual input are represented by populations of neural units that have broad selectivity for the preferred motor command and visual hand position. The network is learned by the backpropagation algorithm. In this section, the overview of the model is described. Details of the model are explained in the Appendix.

**Figure 3:**
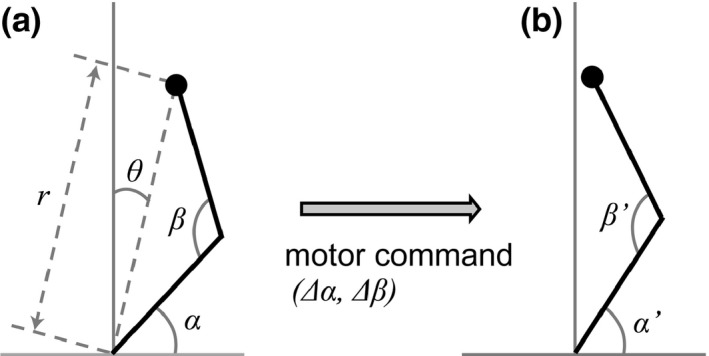
(a) The arm is modeled by two‐joint limbs with joint angles *(α, β)*, and the visual feedback of the hand position is obtained in polar coordinates *(r, θ)*. (b) The arm is controlled by the motor command of the joint angle displacement *(Δα, Δβ)*. After the movement, the joint angle is updated to *(α′, β′)* = *(α + Δα, β + Δβ)*

The arm is modeled by two‐joint limbs moving in the horizontal plane driven by motor commands in the form of relative displacements of the joint angles (Figure 3). The motor commands are randomly generated from a uniform distribution in a certain range during the learning phase. The endpoint of the hand is observed through vision. The visual signal is represented in polar coordinates that consist of the azimuth from the body axis and the distance from the body center (Figure 3a). Infants that are at least 5 months old are sensitive to binocular information for depth (Gordon & Yonas, 1976). In the present model, we adopted the assumption that depth perception is already developed. For simplicity, the body width is neglected and the shoulder joint is placed at the center of the body.

The network consists of two streams of three‐layered artificial neural units. Each stream learns the forward and inverse transformations (Figure 2). The forward network learns the transformation from a motor command to a visual hand position, which is the consequence of the motor command. In order to predict the visual hand position after the movement, the visual hand position before the movement is necessary. The network was provided with the visual hand position before the movement as input. At the same time, the forward network has an internal recurrent connection in its hidden layer, enabling the network to internally maintain the information about the current hand position. In the simulation, visual feedback is sometimes unavailable at a certain rate. This reflects the fact that an infant does not always look at its hand. The lack of visual feedback helps the network to learn to generate an internal representation of the hand position.

The inverse network learns the transformation from a visual hand position to a motor command, which is required to achieve the hand position. The information about the hand position before the movement is provided by the connection from the hidden layer of the forward network. Although this scheme provides a ‘retrospective’ mapping of visual and motor information, it only reframed the sensorimotor mapping that was implemented in the DIRECT model (Bullock et al., 1993). The CNS does not incorporate the causal relationship between motor commands and vision; it only maps the sensorimotor contingency.

**Figure 4:**
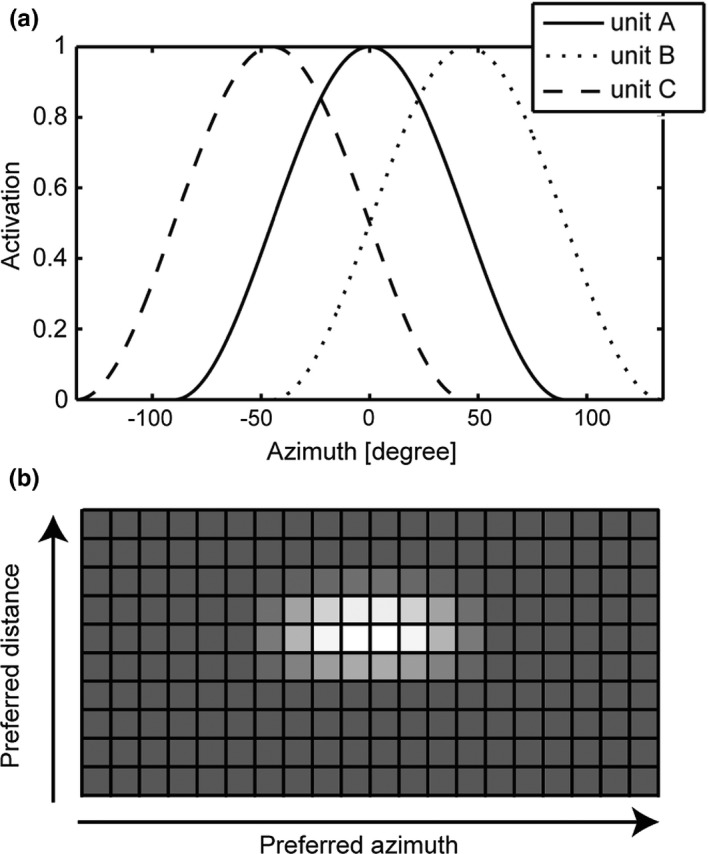
(a) Examples of visual unit that have preferred azimuths and a broad sensitivity (90 degrees). The preferred azimuths of units A, B, C are 0, 45 and −45 degrees, respectively. (b) An example of neural population coding of a visual hand position. Each cell represents the activation level of units of the visual input layer. A greater amount of white indicates higher activation. The position of the cell is arranged in the order of preferred distance and preferred azimuth of each unit. This example is for the case in which the visual input is at half the distance of the arm length and where the visual angle is 0 degrees in azimuth. The cells located around the center, whose preferred distance is around 1 and preferred azimuth is around 0 degrees, show high activation

When the motor command and the visual hand position are input to the network, they are represented by populations of motor or visual units in the place code. Each unit has preferred joint displacements (motor commands) or hand positions (vision) and broad selectivity. In Figure 4a, examples of visual units that have preferred azimuths and a broad sensitivity (90 degrees) are shown. Neural unit A has a preferred azimuth at 0 degrees, that is, this unit is activated the most when the visual hand position is placed at 0 degrees in azimuth. Unit A also shows weak activation when the hand position is far from 0 degrees in azimuth due to broad selectivity. Unit B and C have preferred azimuths at 45 and −45 degrees, respectively. When the hand position is at 0 degree in azimuth, although unit A exhibits the highest activation, units B and C also show activation to some extent. Visual units have this kind of sensitivity for distances of the hand position as well. By preparing many visual units with slightly different preferred azimuths and distances, the visual hand position is represented ambiguously in the population (Figure 4b). The motor units also have broad selectivity and preferred joint angle displacements both for the elbow and shoulder. The tuning curve of the sensitivity is determined by a cosine function, which minimizes the influence of the noise (Todorov, 2002).

The motor command generated by motor babbling is input to the forward transformation network, and the signal propagates through hidden units to form an output visual representation. Units in the input, hidden, and output layers exhibit activation that ranges from 0 to 1. The activation of the units in the input layer represents the population‐coded motor command described above. The units in the hidden layer have connections with all motor input units, the hidden units (recurrent connection), and the visual input units (for the hand position before the movement). The hidden units receive the signal from all the connected units. The units in the output layer have connections with all the hidden units. The activation of the pre‐synaptic units is multiplied by the connection weights and is input to the post‐synaptic units. The post‐synaptic units conduct a summation of the weighted activation of the pre‐synaptic units and scale the summed signal to the activation ranging from 0 to 1 in a monotonic but nonlinear manner (with a logistic function). The visual representation of the output layer is compared to the visual representation of the actual hand position, which is a consequence of the motor command, and the error signal is used to update the network connection weights following the backpropagation algorithm (Rumelhart, Hinton, & Williams, 1986). At the same time, the population‐coded visual hand position after the movement is input to the inverse transformation network and propagated through the hidden (inverse) layer to the output motor representation. Note that the hidden layer in the inverse network has a connection from the hidden layer in the forward network. The output is compared to the representation of the motor command, which is generated to move the hand to the position just input to the inverse network, and the error signal is used to update the network connection (Figure 2).

## 3 SIMULATION

### 3.1 Learning of the network

The network was learned following the procedure described above, and several tests of forward and inverse transformations were conducted. In the simulation, the numbers of neural units in the motor input layer, the visual input layer, and the hidden layer were 100, 200, and 400, respectively. A unit in the motor input layer has one of 10 different preferred angle displacements for each elbow and shoulder joint. Similarly, a unit in the visual input layer has one of 10 different preferred distances and one of 20 different preferred azimuths. The preferred vector is arranged at regular intervals to cover twice the size of the definition range of the vector. This means that some units have their preferred vector outside of the definition range. These out‐of‐range preferred vectors are necessary for the neural population to represent the edge of the vector space. Units that have the preferred vector slightly inside the edge also respond to the input vector just at the edge; consequently, the vector decoded from the neural population shifts to the center of the space. Units with out‐of‐range preferred vectors allow representation of the vector at the edge.

The range of motor command is −20–20 degrees for each joint, and the visual range is 0–3 for distance and −90–90 degrees for azimuth, centered at the sagittal plane. Note that the length of each upper and lower arm is set to 1; therefore, the maximum reaching distance is 2, and the position at distances greater than 2 could be defined as the out‐of‐reach area. The range of joint motion is 0–180 degrees for both the shoulder and the elbow (see Figure 3). When the motor command moves the joint out of range, the joint will stop at the edge of the range. The visual availability rate is 0.8, meaning that visual feedback is unavailable in 20% of the learning iterations.

The learning rate of the backpropagation was fixed to 0.05. We did not use a momentum term, a Falman offset, or a bias node, because our intent was to focus only on the availability of the network as the developmental mechanism, not on the quality of the network learning.

### 3.2 Tests for the forward and inverse transformations

After 1,200,000 instances of learning iterations, we tested the network's ability to generate forward and inverse transformations. Before each test, the network state had to be initialized by iterating the network propagation several times and fixing the visual feedback and motor command, respectively, to the initial hand position and zero. This meant that the hidden layer in the forward transformation network would assume the internal representation of the initial hand position. We carried out 20 iterations for this initialization.

**Figure 5:**
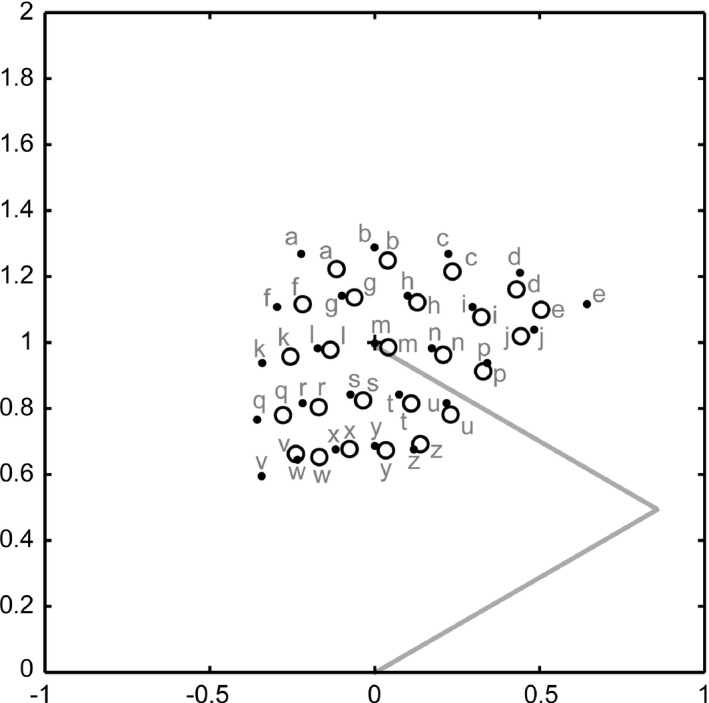
Results of the forward transformation test. The actual and predicted hand positions after movements are illustrated as black dots and circles in the Cartesian space, respectively. Alphabetic labels denote correspondence. Gray solid lines represent the arm in the initial configuration

The forward transformation test examined the predicted visual feedback when several different motor commands were input into a network whose state was set to represent the initial hand position. The initial hand position was set to *(r, θ) = (1, 0)* (i.e., *(α, β) = (30, 60)* and *(0, 1)* in Cartesian space), and the motor commands for the test were 25 combinations of *Δα = {−20, −10, 0, 10, 20}* and *Δβ = {−20, −10, 0, 10, 20}*. Figure 5 shows the resultant actual hand position and the predicted hand positions decoded from the neural representation ***y*** ^*^ *(t + 1)* in the output layer. The outputs of the forward transformation approximately predicted the visual input of the hand position after movement, although the prediction was comparatively inaccurate for the motor commands at the limits of the range.

**Figure 6:**
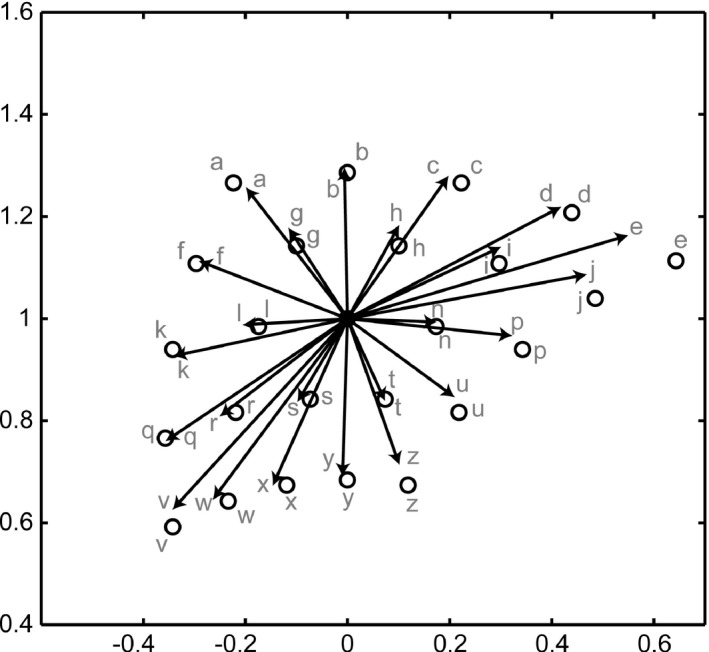
Results of the inverse transformation test. The ‘target’ positions and output motor commands are illustrated as circles and black arrows in the Cartesian space. Alphabetic labels denote correspondence

The inverse transformation test examined the motor commands generated when the inputs into the settled network representations of visual positions were different from the actual hand position. During learning, the visual input was always derived from the hand position of the self after the movement. The movement had no target. Here, if the input was a new visual representation that was not the position of the hand, the inverse transformation network would interpret this position as the hand position after the movement. It would then generate a motor command to achieve the movement toward the new visual input, that is, a ‘target’ position. The initial hand position in this test was *(r, θ) = (1, 0)*, and the target positions were the hand positions that would be achieved when the motor commands of 25 combinations of *Δα = {−20, −10, 0, 10, 20}* and *Δβ = {−20, −10, 0, 10, 20}* were output. Figure 6 shows the target positions and motor commands decoded from the output layer ***u*** ^*^ *(t)* of the inverse transformations. The outputs of the inverse transformation moved the hand approximately toward the target positions, although the motor commands were comparatively small for the target at the limits of the motor command range.

### 3.3 Simulations of reaching and pointing

**Figure 7:**
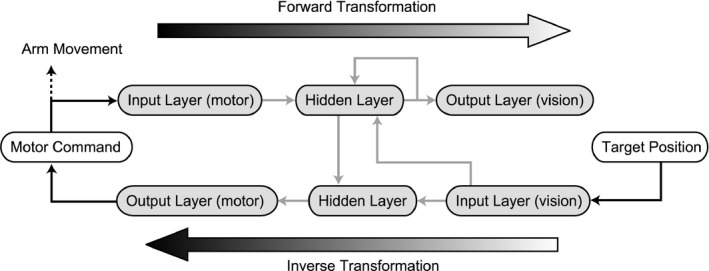
Network structure for reaching simulation by a loop of the forward and inverse transformations. The output of the inverse transformation is input to the forward transformation

For the inverse transformation test, we placed the targets at distances that were reachable in a single step of the transformation. However, as mentioned in the introduction, infants can reach for a target using a loop of forward and inverse transformations, even in the dark. To simulate this ability, we input the output of the inverse transformation to the forward transformation (Figure 7). Note that visual feedback from the hand was not used in the simulation, making this equivalent to a movement performed in the dark (see Clifton et al., 1993).

**Figure 8:**
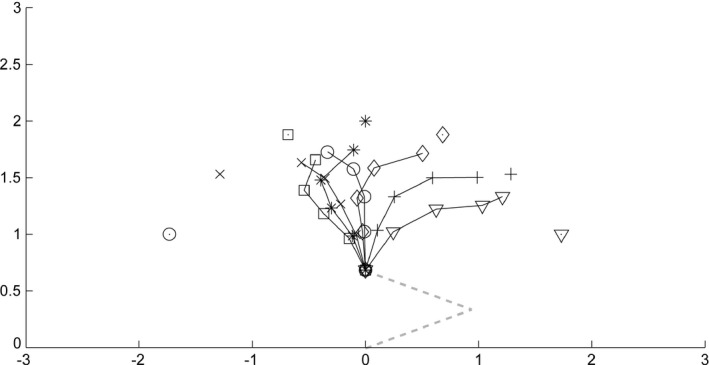
Hand trajectories for targets placed at a reachable distance in the simulation of reaching. A circle, an ‘x’, a rectangle, an asterisk, a diamond, a cross, and a triangle denote targets for reaching, and these marks linked with solid lines denote corresponding hand trajectories. The dashed line represents the initial arm configuration

Figure 8 shows the trajectory of the hand generated by the loop of the transformations with input from seven targets. We placed the targets at a distance of 2 (i.e., just at the reaching distance), with an initial arm configuration of *(α, β) = (20, 40)*. The transformations were iterated four times. Because the distance traveled by a single motor command issued during learning is much smaller than the movement distance required in the reaching simulation, the network needs to generate successive motor commands to achieve reaching. The loop of the forward and inverse transformations successfully generated the sequence of motor commands toward the target, especially for a target in the ipsilateral space.

**Figure 9:**
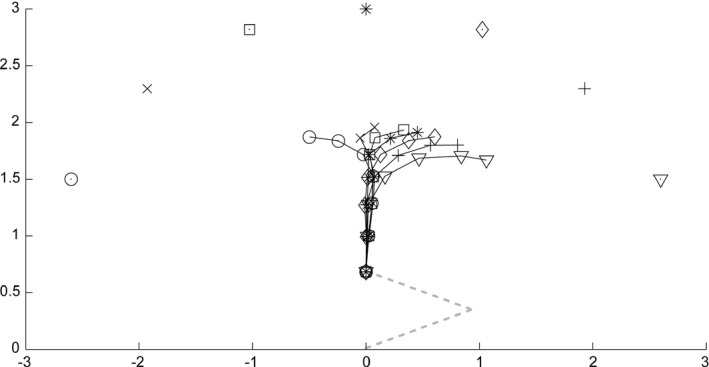
Hand trajectories for targets placed at an distance in the simulation of reaching. A circle, an ‘x’, a rectangle, an asterisk, a diamond, a cross, and a triangle denote targets for reaching, and these marks linked with solid lines denote corresponding hand trajectories. The dashed line represents the initial arm configuration

Even when the targets were placed at an out‐of‐reach distance, the model reached toward the targets (Figure 9). The network was initialized to attain the same state as in a simulation toward a target at a within‐reach distance. The target distance was set to 3, and the loop of the forward and inverse transformations was iterated seven times. The resultant trajectories of the hand first ran approximately straight ahead, and they then deviated toward each target. Note that the network was not provided with the information about the arm length or the reaching distance, and it had not experienced visual input derived from the out‐of‐reach position during learning. Although the resulting reaching trajectories were biased rightward, and reaching toward the lateral targets fell in less lateral positions, observers of the behavior would understand that the behavior was oriented toward the targets. This emergence of arm extending behavior toward an out‐of‐reach target might be based on the development of pointing.

## 4 DISCUSSION

### 4.1 Emergence of reaching and primitive pointing action without social intention

The present model simulated emergent reaching toward a visual position at a within‐reach distance and an out‐of‐reach distance. During the simulation of reaching, the vision of the hand was removed. This means that the vision of the hand is not necessary for executing reaching, although it is necessary during learning, which is consistent with the observation noted by Clifton et al. (1993).

The simulation results showed that reaching toward a target placed in the contralateral space (the left side of the midline, because the right arm is modeled) is less accurate than in the ipsilateral space. This is mainly because the shoulder joint has to be flexed to reach the targets in the contralateral space, despite a relatively extended initial posture, while the elbow joint has to be fully extended for all of the targets. The targets in the contralateral space are further from the initial configuration in the joint space. This difficulty of reaching toward contralateral targets is also seen in infant behavior. Infants first contact objects in front of the ipsilateral shoulder, and between 9 and 17 weeks, there is an increase in reaching that crosses the midline (Provine & Westerman, 1979).

Even in the ipsilateral space, the model's output did not accurately point to the target. However, the ‘orientation’ (see Carpendale & Carpendale, 2010) was accurate, due to constructing the relationship between the pointing action and the referent object through learning. Our current model suggests that infants would start their inaccurate pointing‐like behavior before they accomplish an accurate pointing action, and it also revealed one of the mechanisms underlying the emergence of pointing behavior.

The emergent action would help infants to obtain some kind of feedback (e.g., finger tactile) from the environment, and infants would discover the relationship between the action and the consequent feedback. If this resultant feedback of the action is a motivational force of infant behavior, the presented object that would lead infants to expect the (tactile) feedback when they reach toward it can be a ‘target’ of intentional reaching behavior. In the case of pointing, observers (e.g., parents or caregivers) of the action will provide social feedback to the infant in some manner. This reaction of the other person will then become the aim of the action, and communicative intention will ultimately emerge. Although the manner in which the reaction is sublimated to the intention remains unclear, the proposed model explains the emergence of actions that trigger reactions.

The current model did not include extension movement of the index finger, which plays a key role in typical pointing. While merely reaching toward an out‐of‐reach target without finger extension can be criticized as not being a referent behavior that indicates communication, Blake, O'Rourke, and Borzellino (1994) have reported ‘reach out’ gestures where infants reach toward out‐of‐reach targets without finger pointing, as we have simulated in the present study. Therefore, we could argue that this kind of reaching also appears to have communicative intentions (for review, Leavens & Hopkins, 1999).

### 4.2 Reference frames and neural representations

The proposed model acquired sensorimotor transformations between a visual position in body‐centered polar coordinates and a motor command in joint space. Pointing behavior performed in the dark in adults incurs a pointing error that varies systematically with the azimuth of the target (Soechting & Flanders, 1989; Yoshida & Inui, 2004). This indicates that the target of pointing is represented in body‐centered polar coordinates. The current result of pointing simulation showed that pointing to lateral targets fell in a less lateral position, as it did in adult pointing. Because signals in the early stage of the visual process are represented in a retinotopic reference frame, target representations have to be transformed between retinotopic and spatial reference frames, and neural network models with the ability to learn this transformation have been proposed (Xing & Andersen, 2000; Zipser & Andersen, 1988).

The visual inconsistency was simulated by preventing visual feedback of the hand during the learning period. Although the actual biological system suffers from intrinsic neural noises, we did not implement them in the present model because we cannot determine the level of noise and where they interfere in the model's simulation. These noises might affect the form of neural coding. The cosine tuning, which is predetermined in the present model, is reported as an effective way to compensate for neural noise (Todorov, 2002).

### 4.3 Advantages of the proposed model

The dynamical system as a motor development model was proposed especially in the context of cyclic movement, such as stepping (Kamm, Thelen, & Jensen, 1990) or crawling (Kuniyoshi & Sangawa, 2006). Although the dynamical system is well designed as a pattern generator, it does not explain how discrete ‘orienting’ movements such as reaching and pointing develop. A neural network model is more appropriate for accommodating the relationship between input and output in a movement that has a target.

Schlesinger, Parisi, and Langer (2000) proposed a neural network model for infants’ reaching development using genetic algorithm‐like ecological networks with unsupervised learning. Their model has succeeded in acquiring reaching toward a visually presented object without the explicit representation of the ‘error’ between the target object and the hand position. Instead, the tactile sensation of the contact of the hand with the object acts as the driving force for the network to learn reaching toward the object. However, the acquired reach was not the direct reach from the initial hand position to the object; rather, it was a two‐step reach where the hand was brought back to the position near the body first before traveling toward the object. Although early infants show indirect (curved) reach (Mathew & Cook, 1990), this kind of ‘returning’ behavior was not observed in the present study. Moreover, the network proposed by Schlesinger et al. (2000) can only learn reaching toward touchable objects, not objects at an out‐of‐reach distance.

Our proposed model spontaneously acquired a non‐circular reaching action toward targets at within‐reach and out‐of‐reach distances by the forward and inverse transformation loops and broadly tuned motor and visual representations.

### 4.4 Limitation of the current model and future model implementation

The forward transformation in the present model is in line with Elman's (1990) notion of Simple Recurrent Networks (SRNs). However, our model did not learn the sequence of motor commands, while an SRN has the ability to achieve sequential learning. In the future, by implementing infants’ intention of reaching, which requires a motor sequence, the implemented SRNs in the forward transformation may help the model learn more sophisticated motor sequences, such as those exhibited by infants after the age of 9 months (Bushnell, 1985).

When reaching toward and pointing at objects in three‐dimensional (3D) space with one arm, seven degrees of freedom (DoFs) of the shoulder, elbow, and wrist joints were used. The control problem always involves redundant DoFs. While computationally solving the problem of DoFs is an important issue, how humans (or animals) solve this is still being debated. Therefore, for simplicity, our proposed model only moves in two‐dimensional (2D) space with two control parameters. In the future, we would like to solve this problem by introducing mechanical (Hirashima & Oya, 2016) or neural (Todorov, 2000) constraints.

During the model's learning, the only visual input was the infant's hand. However, in real life, objects or others’ hands may also appear in infants’ visual fields. Infants may develop the agency of the visual movement through sensorimotor contingency (Pitti, Mori, Kouzuma, & Kuniyoshi, 2009). Alternatively, the contingency may tell the learning system which visual input should be related to the motor command, and acquired visuomotor transformations may be used for recognition of the agency.

Although the proposed model explained the emergence of arm extending behavior toward targets at out‐of‐reach distances, how the social meanings of the behavior are acquired was not modeled here. We speculate that infants discover the social meanings of the behavior through two steps: First, they will acquire this meaning by touching the object at the end of the reaching movement, wherein they will determine the relationship between the movement and the resultant tactile sensation. The contingency of the movement and the sensation may reinforce the behavior, and the richness of the sensory stimulus when infants manipulate the object would motivate them to reach toward the visual input generated by the object. This would make visually guided reaching within the reaching distance more frequent. Second, if people who observe an infant trying to reach toward out‐of‐reach objects bring the objects to the infant, the resultant tactile stimulus may also motivate the behavior and help infants to use it as a communicative tool to satisfy their motivation. In order to verify these possibilities, experiments involving the longitudinal observation of infants and their caregivers would be helpful in uncovering the order of emergence of behaviors such as reaching, touching, bringing, and pointing (Carpendale & Carpendale, 2010).

## 5 CONCLUSIONS

We proposed a neural network model that learns forward and inverse sensorimotor transformations and acquires reaching control using these transformations. Sensory inputs and motor commands are coded in place coding with neural units that have broad sensitivities, and the network is learned through motor babbling. Even in the absence of targets during learning, the learned network successfully simulated arm extending action toward targets located at a within‐reach and out‐of‐reach distance. The ability of the proposed model to perform arm extending toward out‐of‐reach targets might be a basis for reaching and pointing behaviors that contain communicative intentions, which leads to social behaviors.

## APPENDIX 1

### Arm configuration, motor command and vision

The proposed neural network model learns relationships between motor output and visual input of the hand. The arms are modeled with a two‐joint link rotating in a horizontal plane. Consider that an arm configuration ***x**(t) = (α, β)* at time *t* is updated by a motor command ***u**(t) = (Δα, Δβ)*, and that the resultant arm configuration is ***x**(t + 1) = (α′, β′)*. The hand position is observed visually in polar coordinates. Given that the visual feedback at time *t* is ***y**(t) = (r, θ)*, the forward transformation predicts visual feedback ***y**(t + 1)* from the motor command *u(t)*, and the inverse transformation calculates the motor command ***u**(t)* that is necessary to achieve the hand position ***y**(t + 1)*.

### Network structure

The proposed model consists of two streams of three‐layered neural networks to bring about learning of the forward and inverse transformations (Figure 2). In the forward transformation network, the motor command ***u**(t)* is input, and the prediction of the visual feedback ***y*** ^***^ *(t + 1)* is output. The visual feedback ***y**(t)* is also input into the forward transformation because information on the hand position ‘before’ the motor command ***u**(t)* is necessary for prediction of the hand position ‘after’ the movement. The hidden layer in the forward transformation has a recurrent connection. At time *t*, the activation of the hidden layer in the forward transformation, denoted by ***h***_*f*_ *(t)*, contains internal intermediate representation of the hand position for the prediction of the visual feedback ***y*** ^***^ *(t)*. Therefore, ***h***_*f*_ *(t)* has the hand position information before ***u**(t)* is generated. The recurrent connection can be used as an alternative to the visual input ***y**(t)* before the movement, enabling the network to work even in a case where visual feedback is unavailable. Note that in the actual network, the motor command ***u**(t)* and visual feedback ***y**(t)*, respectively, are encoded into the neural representations ***u*** ^*~*^ *(t)* and ***y*** ^*~*^ *(t)*.

For the inverse transformation network, the visual feedback after the movement, ***y**(t + 1)*, is input, and the ‘postdiction’ ***u*** ^***^ *(t)* of the motor command to achieve the observed hand position is output. The activation of the hidden layer in the forward transformation, that is, ***h***_*f*_ *(t)*, is also input to the inverse transformation in order to provide information about the hand position before the movement.

### Motor and visual representations

The motor command and the visual feedback are encoded by a population of units that have broad selectivity for the preferred joint angle displacement and the preferred hand position, respectively. The selectivity of each unit is cosine tuned; that is, given that the ***R*** ^*N*^ vector to be encoded is ***z** = {z*_*j*_ *| j = 1,…,N}*, and that the unit *i* has the preferred vector ***p***_*i*_ *= {p*_*ij*_ *}*, the activation *a*_*i*_ of the unit *i* is defined by:

$$\begin{array}{cc} a_{i}=\Pi_{j}\left[0.5\;cos(2\pi \;\right|z_{j}-p_{ij}\left|/W)+0.5\right] & \text{if}\;|z_{j}-p_{ij}|\;<W/2\;\text{for}\;\text{all}\;j,\\ a_{i}=0 & \text{if}\;\text{otherwise.} \end{array}$$

Note that *| ‐ |* denotes absolute value, and *W* is the size of the possible range of the encoded vector. In the cosine tuning, the activation of unit *i* will be large (up to 1) when the input vector ***z*** is close to the preferred vector *p*_*i*_, and the unit will show weak activation even when the vector input vector deviates from the preferred vector. *Π* denotes the production of each element. An example of the activation of visual units is shown in Figure 5, where it is demonstrated that the neural units that have preferred vectors around the input vector are more highly activated. Here, we do not mean that the positions of the neurons in the actual brain are arranged in the order of preferred vectors; they are depicted in this way in Figure 5 simply for easier understanding.

### Network propagation and learning algorithm

The input signal propagates through the network following:

$$a_{k}=1/\left(1+exp\left(\sum_{l}w_{kl}\;a_{l}+\theta_{k}\right)\right)$$

where *a*_*k*_ denotes the activation of unit *k* in the hidden or output layer; *a*_*l*_ denotes the activation of unit *l*, which has a connection to unit *k*;* w*_*kl*_ denotes a connection weight from unit *l* to unit *k*; and *θ*_*k*_ denotes an activation threshold of unit *k*.

The learning procedure is as follows: (1) Initialize the connection weights of the network to a small random value; (2) initialize the activation of the hidden units in the forward transformation to a small random value; (3) set the initial arm configuration ***x**(t)* and calculate the visual observation ***y**(t)*; (4) generate a random motor command ***u**(t)*; (5) input the encoded ***u*** ^*~*^ *(t)* and ***y*** ^*~*^ *(t)* into the forward transformation network to obtain ***h***_*f*_ *(t + 1)* and output ***y*** ^***^ *(t + 1)*; (6) move the arm by ***u**(t)* and obtain the next arm configuration ***x**(t + 1)*; (7) obtain visual observation ***y**(t + 1)*; (8) input the encoded ***y*** ^*~*^ *(t + 1)* and ***h***_*f*_ *(t)* into the inverse transformation network and obtain output ***u*** ^***^ *(t)*; (9) calculate the output error between ***y*** ^***^ *(t + 1)* and ***y*** ^*~*^ *(t + 1)*, and ***u*** ^***^ *(t)* and ***u*** ^*~*^ *(t)*; (10) change the connection weights and the thresholds following the backpropagation algorithm; and (11) generate the next motor command ***u**(t + 1)* and iterate the procedure from (5).
